# How Do Children Reason About Mirrors? A Comparison Between Adults, Typically Developed Children, and Children With Autism Spectrum Disorder

**DOI:** 10.3389/fpsyg.2021.722213

**Published:** 2021-10-21

**Authors:** Alessandro Soranzo, Marco Bertamini, Sarah Cassidy

**Affiliations:** ^1^Department of Psychology, Sociology and Politics, Sheffield Hallam University, Sheffield, United Kingdom; ^2^Department of General Psychology, University of Padova, Padua, Italy; ^3^Department of Psychology, University of Liverpool, Liverpool, United Kingdom

**Keywords:** autistic spectrum disorder, reasoning, children, perspective-taking, theory of mind, reasoning about mirrors, typical and atypical development

## Abstract

The information about what one can see and what other people can see from different viewpoints is important. There are circumstances in which adults and children make systematic errors when predicting what is visible from their own or others’ viewpoints. This happens for example when reasoning about mirrors. We explored differences among three developmental groups: young adults (*N*=60) typically developing children (*N*=30); and children with autism spectrum disorder (ASD, *N*=30). We used an illustration of a top-down view of a room with a mirror on a wall (Room Observer and Mirror Perspective test: ROMP). Participants selected (circled on paper) which objects behind the observer in the room were visible, reflected from the mirror and from a given position (viewpoint). For half of each group, the observer in the room was described as a teddy bear; for the other half, it was described as a child. Overall, there were many errors in all groups, which we separate in errors of ignoring the viewpoint (same response to all three locations) and inversion errors (choosing objects on the left instead of the right or vice versa). In addition to the overall task difficulty, the ASD group made relatively more mistakes of ignoring the viewpoint compared to the other groups and underestimated how many objects were visible in the teddy bear condition that is when the viewpoint was an inanimate object. We suggest that this is related to a delay in theory of mind (ToM) development.

## Introduction

As an organism moves in the environment, the view changes, revealing or hiding different objects. Taking into consideration, the viewpoint of other individuals is also important because it determines what someone can see at any point in time. Research has found that when people evaluate a scene in terms of what is visible to an individual, they make random and systematic errors. Children often display an egocentric bias (Piaget and Inhelder, 1956; Moore et al., 1995), and errors in both children and adults can be influenced by the ability to distinguish our own perspective from the perspective of others (Moore et al., 1995; Birch and Bloom, 2004; Epley et al., 2004). In a complementary fashion, the importance of other people’s viewpoint is also highlighted by the suggestion that adults compute other perspectives even when it is not necessary or detrimental to the task (Samson et al., 2010; Wilson et al., 2017).

A situation that is particularly challenging for both adults and children is that of a mirror reflection. The general pattern is one in which people claim that what is visible in a mirror are the objects in front of the mirror itself, irrespective of the location of the viewer (Croucher et al., 2002; Bertamini et al., 2010). For example, when asked to judge where a person entering a room and moving parallel to a mirror surface would see their own reflection a large percentage of adults believe that it is not necessary to reach the edge of the mirror (Croucher et al., 2002). Some (a minority) believe that the image will appear at the far edge rather than the near edge (Bertamini et al., 2003). Similar errors are found for more complex movements with respect to the mirror surface (i.e. not parallel motion, Savardi et al., 2010).

**Figure 1:**
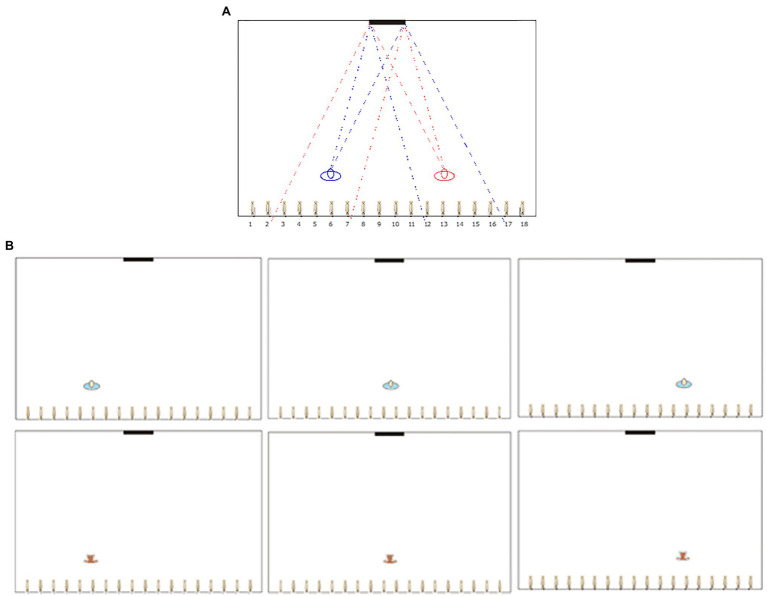
**(A)** The Room Observer and Mirror Perspective test (ROMP) task is based on the diagram of a room containing an observer and a mirror. The task is to select what objects on the back wall would be visible to the observer. For illustration, we overlap two observers. For the observer on the right, the visible sweets are 3–7; for the observer on the left, the visible sweets were 12–16. **(B)** Six layouts used in this study. Each diagram was printed on A4 paper. The columns show the three locations (left, middle and right), and the rows show the two observers (Child, Teddy bear).

The root of the difficulty is that people fail to understand the role of the viewpoint. This is clearly illustrated by the Room Observer and Mirror Perspective (ROMP) task (Bertamini et al., 2010; Bertamini, 2014; Bertamini and Soranzo, 2018). Here, individuals are asked to decide which objects along a wall of a room are visible to a person facing in the opposite direction, given that a mirror is present on the wall facing the person. The procedure is based on a top-down map of a room and a drawing showing the position of the person and of the mirror. When the position of the observer is to the left of the mirror, they could see the objects to the right, and when the observer is to the right, they could see the objects to the left. An example of a diagram used in the ROMP task is shown in Figure 1.

To answer questions about what another person can see individuals need to be able to take someone else’s perspective. The ability to represent that another person sees something different from what we see is referred to as Level-1 visual perspective-taking. By age 2, children understand the role of visual perspective when instructed to show another person a picture (Lempers et al., 1977). Even younger children show sensitivity to gaze information. For example, Brooks and Meltzoff (2002) found that 12-month-old infants followed a person’s gaze direction more when the person’s eyes were open, and Sodian et al. (2007) found differences in looking time in 14-month-old infants, showing that they were aware that a person could or could not see a target object. The ability to understand how another person sees things and how objects appear to them is referred to as Level 2 perspective-taking. Although Level 2 may be achieved later than Level 1, they both appear present in the first few years of life in humans (Flavell et al., 1981; Moll and Meltzoff, 2011).

Individuals who have a delayed or atypical ability in perspective-taking may find perspective-taking tasks more difficult. This is the case of children with a diagnosis of autism spectrum disorder (ASD). We briefly discuss theory of mind (ToM) in relation to ASD. ToM includes the ability to process another’s perspective and enable individuals to comprehend and predict the behaviour of others (Baron-Cohen et al., 1985). Tasks used to explore ToM involve perspective-taking (Reed and Peterson, 1990). It has been shown that children with ASD demonstrate deficits in perspective-taking tasks as compared to age-matched typically developing children (Rehfeldt et al., 2007). Individual differences in cognitive ability impact on performance in these tasks (Hughes et al., 2005; Ronald et al., 2006; Conway and Bird, 2018), such as intelligence, language acquisition and developmental disorders. Where some research indicates that ToM is a single cognitive ability, which is either acquired or not (Happé, 1994; Hughes and Ensor, 2007), other research suggests that this acquisition is a process, achieved in a series of developmental stages (Wellman, 2002; Wellman and Liu, 2004). Children understand belief and desire, but the understanding of false belief is more difficult to acquire (Harris, 1992; Leslie, 1994). This difficulty may lie in the inability to inhibit the natural instinct to respond from one’s own perspective (Moore et al., 1995; Carlson and Moses, 2001).

Our study is not specifically about ToM, and we have described it because it is relevant to understand possible individual differences. A child is typically ascribed to have achieved ToM by age 4, although this process continues up to around age 11 (Perner and Wimmer, 1985; Stone et al., 1998; Liddle and Nettle, 2006; Apperly et al., 2009). If a ToM is normally developed by age 11, it follows that typically developing children should be as competent as adults in visual perspective-taking tasks by this age.

There is an association between autism and ToM (Baron-Cohen et al., 1985; Heavey et al., 2000). ASD children may perform poorly on perspective-taking tasks (Birch and Bernstein, 2007). However, if ToM is not inextricably linked to the ability to understand visual perspective, then children with a developmental delay should perform similarly to children who are typically developing. Children and adults with ASD generally perform well on visual tasks (Mottron et al., 2006; Tager-Flusberg, 2007), and this may also account for similar or indeed superior performance on some spatial tasks.

## The Study

In this study, we used a diagram of a room, and the task was to indicate what an observer in the room could see when looking at a mirror on a wall (ROMP task). In particular, the task was to evaluate which objects (resembling sweets in this case) behind the observer were visible from a mirror (indicated by a thicker line on the wall in front of the observer). Previous studies have found a tendency to overestimate what is visible and a lack of sensitivity to viewpoint (Bertamini et al., 2010; Bertamini and Soranzo, 2018); however, the procedure has not been used with children before. Furthermore, different types of development were not considered before. Here, we focus on performance of typical development children (TD children) children with a diagnosis of autism spectrum disorder (ASD children) and young adults.

In all groups, we used the same stimuli, shown in Figure 1. They were simple diagrams, and each group of participants (adults, TD children, and ASD children) was divided into two subgroups. For one subgroup, the observer in the room was described as a child; for the other subgroup, the observer was described a teddy bear. This manipulation further explores the degree by which individuals can take the viewpoint of another observer and whether it matters if this observer is a human being. We reason that there may be an additional challenge when individuals need to assign a mental state to an inanimate object (a teddy bear). Teddy bears have been previously used to investigate perspective-taking (e.g. Bertamini and Soranzo, 2018; Russo et al., 2018). They have the characteristic that although their semblance suggests that they own a viewpoint, they lack a proper mental state, and it needs to be explicitly assigned.

We therefore hypothesise that it would be more difficult to reason about is visible in the mirror in particular for ASD children and in particular when the viewpoint lacks of a proper mental state.

## Materials and Methods

### Participants

We tested 60 children attending mainstream primary schools in Sheffield (United Kingdom). Thirty had a diagnosis of autism spectrum disorder (ASD group), and 30 were classified as typically developing (TD group). Half of the participants were males, and half were females. The mean age was 9.78years (SD=1.41). Participants were recruited according to whether they had a diagnosis of ASD, and these participants were then matched by the Special Educational Needs Co-ordinator in each school to typically developing children of the same age, sex and ability. The project was approved by the Psychology Research Ethics Panel at Sheffield Hallam University (nr. E7030/2019). For comparison, we also tested 60 young adults. The procedure was the same, but the participants were undergraduate students, enrolled on a Psychology degree. The mean age was 18.63years (SD=1.10). The majority (48) were females.

### Design

The first factor was Development type: there was a group of TD children, a group of ASD children and a group of adults (all TD). Another between-subject factor was the observer in the room (Observer type). For one subgroup, it was described as a child; for the other subgroup, it was described as a teddy bear. Each participant saw three locations of the observer, left, centre and right. We call this within-subjects factor Location (Figure 1).

### Procedure

Data from children were collected in May and June of 2018. Five local schools in North Sheffield were contacted and asked to participate in the study, and four schools responded. Participants were invited to participate in the research *via* a letter to parents, and children were asked for verbal consent at the time of the experiment. Data from young adults were collected during the 2018/2019 academic year.

**Figure 2:**
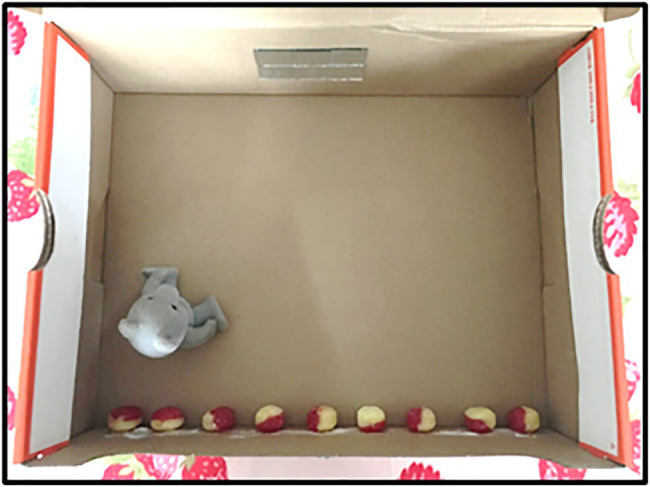
The box was used to illustrate the task. This photo shows a teddy bear, but male and female dolls were also used to illustrate the ROMP task.

Each child was tested individually. Within each group of 30 participants (ASD and TD), 15 (50%) were shown a child, and 15 (50%) were shown a teddy bear. In a familiarisation phase, each participant was shown a 3D model of the room (Figure 2 shows the model with a teddy bear). They were invited to observe either the child or the teddy in each of the three positions and to hold the box to look into it. This was an important stage to ensure that they understood the task and to avoid relying on verbal instructions only, that might have impacted on the performance.

The box was then removed, and the children viewed three separate sheets of A4 paper, showing either a child or a teddy bear in three locations (left, middle and right). Participants were asked to imagine how many sweets could be seen reflected in the mirror, either from the child perspective or from the perspective of the teddy bear. They were informed that the child/teddy could look in any direction, but that it must remain in one place. Children were then asked to circle the number of sweets they believed would be visible in the mirror to the child/teddy. The procedure used for the young adults was the same except that they were not shown the model of the room.

## Results

**Figure 3:**
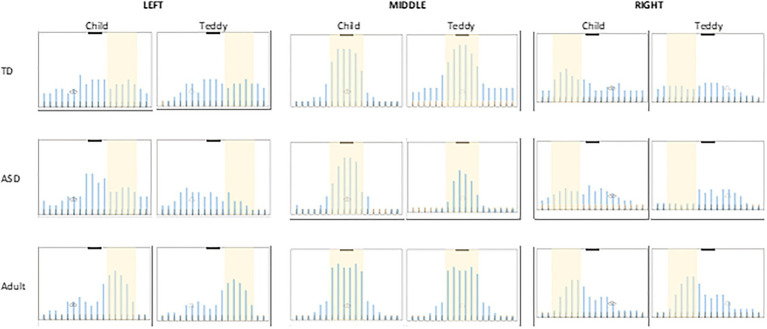
The columns show the three locations (left, middle and right) and the two observers (child, teddy bear). The overall number of times (frequency) each sweet was circled is shown for each condition. The shaded area shows the correct region (visible sweets).

Figure 3 shows the overall number of times each sweet was circled for each condition (e.g. the frequency bar would be 30units if a sweet was selected by all the participants in that condition). The shaded area in each box represents the correct answer. Participants made a large number of errors. If we consider a strict criterion (selecting all the correct sweets and only the correct sweets), no one was perfect, because, as anticipated, the task was difficult.

### Sensitivity to the Viewpoint vs. Sensitivity to Optics

We distinguish between viewpoint sensitivity and sensitivity to optics in the following way. Viewpoint sensitivity refers to the ability to recognise that the location of the viewer matters, even without knowing correctly what is visible from that viewpoint. Therefore, on the basis of viewpoint sensitivity participants should give different answers to different viewpoints (an effect of Location). Sensitivity to optics refers to the ability to recognise that a viewpoint on the left makes objects on the right visible and vice versa. This follows from the optical principle that angle of incidence and angle of reflection are the same, although here we are not interested in explicit knowledge of optics. Note that an individual may know the correct answer based not on knowledge of optics but simply on experience, because when one stands on the right side in a room (as described in Figure 1), they would see objects on the left of the room and vice versa. To assess whether a participant displays these sensitivities, we classified their pattern of responses into three categories: correct, inverted and unbiased.

#### Correct Responses

Participants show a correct pattern of responses when they circled more sweets on the *opposite* side of the viewpoint rather than on the *same* side.

#### Inverted Responses

Participants show an inverted pattern of responses when they circled more sweets on the *same* side of the viewpoint rather than on the *opposite* side. Although this response pattern is wrong, it shows that the participants respond to the location of the viewpoint.

#### Unbiased Responses

Participants show an unbiased pattern of responses when they circled the same sweets for all viewpoint locations. In this case, participants do not consider the location of the viewpoint as a factor, and therefore, there is no bias.

For the sake of clarity, the comparisons of sensitivities were split between adults vs. TD children and TD vs. ASD children. Table 1 presents the frequencies of correct and unbiased responses for the different groups. The *χ*^2^ statistic with Yates’s correction indicates that there is no difference between the two age groups [TD children and adults; *χ*^2^=0.04, *p*=0.84]. However, the comparison within the two groups of children does show an interesting difference. There are more unbiased responses among the ADS children than in TD children. A test of association confirms this pattern: the *χ*^2^ statistic with Yates’s correction was 5.39; *p*<0.05. In addition, there was a tendency for the effect within the ADS group to be larger when Observer type was the teddy bear (correct: 3; unbiased: 7) rather than a child (correct: 7; unbiased: 4), but this pattern could not be statistically analysed due to the small number of subjects per category.

**Table 1:** Correct vs. unbiased pattern of responses in relation to age (left) and in relation to Development type (right).

	TD children	Adults	ADS children
Correct response	14(73.78%)	39(79.69%)	10(47.62%)
Unbiased response	5(26.32%)	10(20.41%)	11(52.38%)

Inverted responses not included.

Our first analysis (Table 1) focused on the correct responses and the unbiased responses (one type of error). In our second analysis, we compared the correct bias (correct responses) and the inverted bias (a different type of error). This comparison provides a measure of sensitivity to optics.

Table 2 presents the frequencies of correct and inverted responses for the different groups. There was no difference in the sensitive to optics between children and adults. A test of association confirms that these frequencies are consistent with the null hypothesis. The *χ*^2^ statistic with Yates’s correction was 2.43; *p*=0.12. There was also no significant difference in the sensitive to optics between the two development types. The *χ*^2^ statistic with Yates’s correction was 0.01; *p*=0.93. Therefore, it seems that the difference that emerged for correct and unbiased responses between the two development types was not due to a difference in sensitivity to optics.

**Table 2:** Correct vs. inverted pattern of responses in relation to age (left) and in relation to Development type (right).

	TD children	Adults	ADS children
Correct response	14(44%)	39(76.47%)	10(52.63%)
Inverted response	11(56%)	12(23.53%)	9(47.37%)

Unbiased responses not included.

Because half of the participants saw an observer described as a child and the other half saw a teddy bear, we lacked the sample size to analyse this difference. These frequencies treat the two cases together. In the next analysis, we take a different approach, we compute a continuous measure of overestimation, and we analyse the difference between the viewpoint type: child and teddy bear.

### Overestimation of What Is Visible

**Figure 4:**
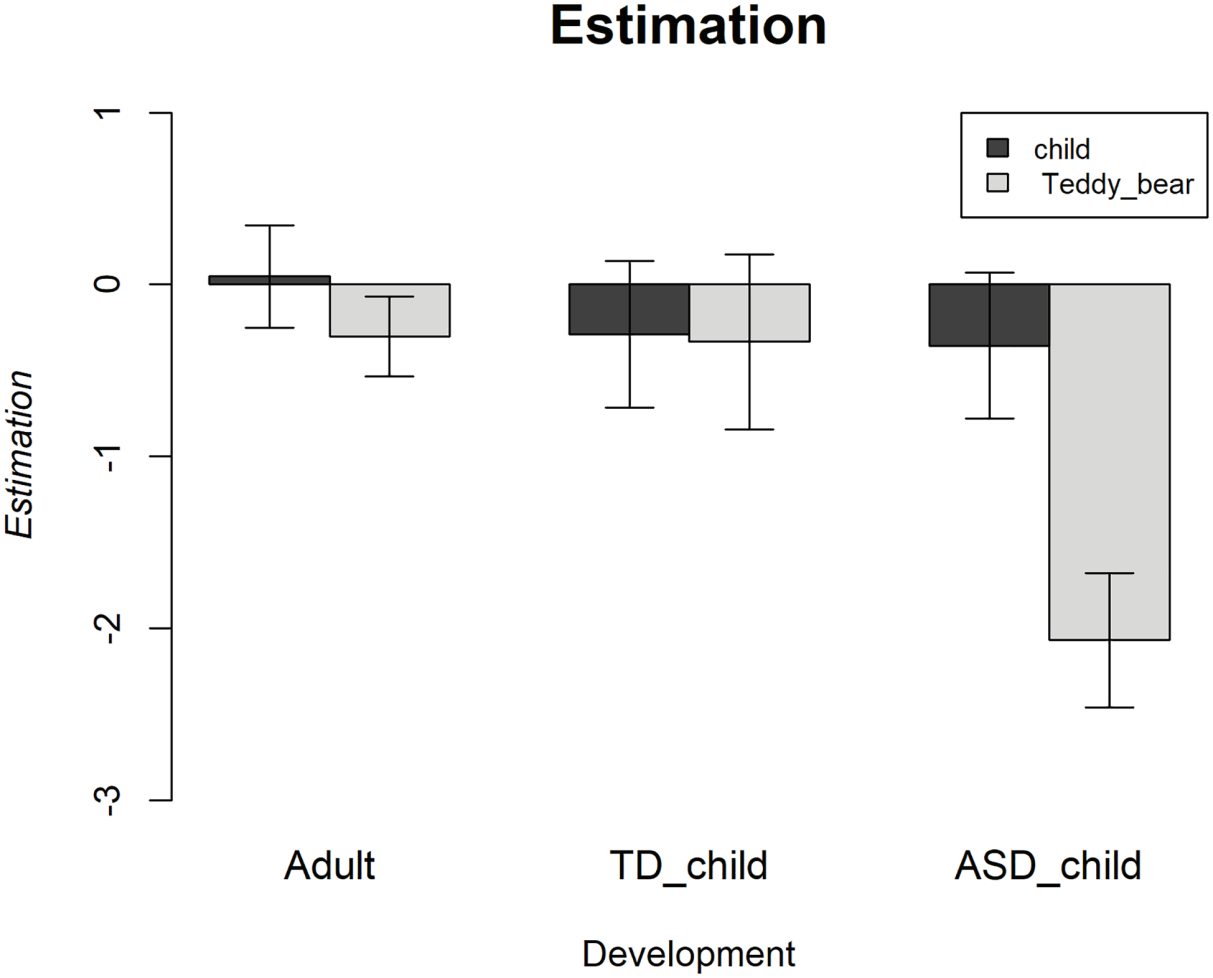
Mean estimation of the visible sweets relative to the correct number. Zero represents a correct answer, while higher values represent an overestimation of how many sweets are visible when looking at the mirror. Error bars are ±1SE for the mean.

To further explore the differences between groups, we analysed the total number of sweets selected minus the correct number of sweets that needed to be circled. Therefore, this index is zero for correct answers, negative values represent an underestimation, and positive values indicate an overestimation of how many objects are visible. Mean estimation values are shown in Figure 4. As a correct number of objects does not imply that the correct items were selected, this analysis focuses exclusively on the quantity of the estimation. It is, therefore, fundamentally different from the previous analyses.

We used generalised linear mixed-effects models implemented in the lme4 package (Bates et al., 2007) in R version 3.5.1 (R Core Team, 2017). Fixed factors were Development (Adults, TD children and ASD children) and Observer (Teddy bear vs. Child). The dependent variable was the number of sweets that the participants selected as visible in the mirror (Estimation analysis). Residuals were independent and normally distributed as indicated by an istrogram plot. The initial random effects structures represented the maximal model (Barr et al., 2013). For both analyses, we included participant as a random intercept with fully crossed random slopes for estimation. The models were simplified until convergence was reached. Log likelihood-ratio (*χ*^2^) comparisons were obtained through the sequential decomposition of the model (Bates et al., 2007), which provided confirmatory tests for the predictors. The marginal and conditional *R*^2^ effect sizes are also reported as measures of the variance explained by the model with the random effect structure included (conditional *R*^2^) and excluded (marginal *R*^2^) from the calculation (Nakagawa and Schielzeth, 2013; Johnson, 2014; Nakagawa et al., 2017).

The maximal models that converged contained random intercepts for participant, but no random slopes. The total explanatory power was substantial (conditional *R*^2^=0.5), and the part related to the fixed effects alone (marginal *R*^2^) was 0.05. The intercept, corresponding to Development type equal to Adult and observer equal to child, was 0.05 [95% CI [−0.79, 0.89], *t*(352)=0.11, *p*=0.912]. Within this model, there were no main effects. However, there was a significant interaction between Development and Observer [beta=−0.42, SE=0.11, 95% CI [−0.64, −0.20], *t*(118)=−3.74, *p*<0.001] although small (std. beta=−0.15, std. SE=0.040). This interaction was further explored through the emmeans R package (Lenth, 2021) which showed a difference between both Adult vs. ASD children (estimated difference=1.7, *p*<0.05) and TS children vs. ASD children (estimated difference=1.7, *p*=0.09) when the observer was the Teddy bear. All other interactions reported a value of *p* >0.84.

The main conclusion from the analysis of estimation is that there was no overall pattern of over or underestimation, as shown in Figure 4. The only significant effect was that of the interaction between Development type and Observer: as shown in Figure 4, this was because of a tendency by ASD children to underestimate what the teddy bear could see.

## Discussion

In this study, typically developed children, children with a diagnosis of autism and adults performed a simple task involving familiar objects. In a top view map of a rectangular room, an observer (a child or a teddy bear) looks towards a wall. On the wall behind them, there are many sweets (ROMP test, Figure 1). What object can they see without turning around and therefore looking at the objects in the mirror?

Despite this simple task and the familiar context, participants made many errors. We distinguish between two types of errors. The original study used this test in young adults (Bertamini et al., 2010) and found that responses were similar for different locations of the viewpoint. This shows a lack of social sensitivity in the sense that the viewpoint of the observer is not seen as relevant. A second type of error happens when the objects selected are on the right side when they should be on the left or vice versa. This shows that the viewpoint is seen as important, but it is used incorrectly. We call this a lack of sensitivity to optics because it shows a lack of knowledge that a location on the left makes objects on the right visible and vice versa. Viewpoint is treated as relevant, but in a way that it is at odds with what is predicted by optics, as well as what is predicted by experience.

To perform this task, subjects need to have an ability to take someone else’s perspective. Since taking someone else’s perspective requires a ToM, we considered the developmental aspects of perspective-taking. Individuals who have a delayed or atypical ability in understanding others’ point of view may find these tasks more difficult. In addition, to further explore the link between ToM and perspective-taking, we consider two types of observers: a human child and a teddy bear (inanimate object; without a proper mental state).

Overall results show that there were many mistakes by both children and adults. This is a task that is simple to describe but participants are unable to locate the exact objects visible. This is consistent with the literature (e.g. Croucher et al., 2002; Bertamini et al., 2003). When we analysed the types of errors, we found no difference in a developmental comparison (TD children and adults). However, when comparing TD children and ASD children, we found a difference specifically for the social sensitivity. For ASD children, there were more responses of the type that ignored the role of the viewpoint. We suggest that this is related to a delay in ToM development (Baron-Cohen et al., 1985).

We also analysed the degree of over or underestimation by comparing the number of objects circled with respect to the correct number. When we compared the groups, children showed a stronger tendency to underestimation if the observer was described as a teddy bear, but this effect was specific to ASD children (an interaction between type of development and type of observer). The pattern for ASD children is consistent across the two analyses, they seem to find the task harder (more errors of ignoring the location of the viewpoint), and they, at least in the case of the teddy bear, tend to underestimate how many objects are visible.

On the one hand, as already noted, this is consistent with our overall hypothesis that being able to understand someone else’s viewpoint is important in this task and may be harder for ASD children. It also seems likely that they struggle with assigning a mental state to an inanimate object. However, there is some evidence of a relative advantage that ASD children have in engagement with anthropomorphic stimuli. They tend to anthropomorphise non-human agents more than controls (Atherton and Cross, 2018).

### Limitations and Future Directions

Although children of both groups belong the same school and were matched also in terms of their ability, a formal assessment of their mental age was not conducted.

It would be interesting in the future to further assess whether performance in the ROMP task varies as a function of the degree of ‘inanimate-ness’ adopted as viewpoints.

### Summary

Overall, this study is a first exploration of developmental aspects of perspective-taking in the context of a mirror task. We note that the ROMP procedure is easy to implement and informative. It also lends itself to compare different agents (animate and inanimate). Our sample was not large, and some of the differences due to development do require confirmation from future work. The motivation for the current study was that the ROMP task requires processing of visual perspective; therefore, we reasoned that a developmental delay may add difficulty to the task. We found that there were more errors for ASD children. We note that it was not a generic pattern of random errors, and they were systematic errors, in particular in relation to the understanding of the role of the viewpoint, precisely the social aspect of the task.

## Data Availability Statement

The original contributions presented in the study are included in the article/Supplementary Material, and further inquiries can be directed to the corresponding author.

## Ethics Statement

The studies involving human participants were reviewed and approved by Psychology Research Ethics Panel at Sheffield Hallam University (nr. E7030/2019). Written informed consent to participate in this study was provided by the participants’ legal guardian/next of kin.

## Author Contributions

AS and MB equally contributed in developing the research question and the project design. They also equally contributed in the statistical analysis and writing-up the manuscript. SC contributed with data collection and literature review. All authors contributed to the article and approved the submitted version.

## Conflict of Interest

The authors declare that the research was conducted in the absence of any commercial or financial relationships that could be construed as a potential conflict of interest.

## Publisher’s Note

All claims expressed in this article are solely those of the authors and do not necessarily represent those of their affiliated organizations, or those of the publisher, the editors and the reviewers. Any product that may be evaluated in this article, or claim that may be made by its manufacturer, is not guaranteed or endorsed by the publisher.
